# Design of a Nutraceutical Gummy Candy Incorporating Hydrolysed Hemp (*Cannabis sativa* L.) as an Antioxidant and Antihypertensive Ingredient

**DOI:** 10.3390/bioengineering12121298

**Published:** 2025-11-25

**Authors:** Álvaro Bastardo, Iván Jesús Jiménez-Pulido, Elena Ordás, Daniel Rico, Nieves Aparicio, Jose María Arjona, Ana Belén Martín-Diana

**Affiliations:** 1 Agrarian Technological Institute of Castilla and Leon (ITACyL), Ctra. Burgos Km 119, Finca Zamadueñas, 47071 Valladolid, Spain; 2 Endocrinology and Clinical Nutrition Research Center (CIENC/IENVA), Department of Medicine, Dermatology and Toxicology, University of Valladolid, Av. Ramón y Cajal, 7, 47005 Valladolid, Spain; 3 Health Research Institute of Valladolid (IBioVALL), 47010 Valladolid, Spain

**Keywords:** hemp, *Cannabis sativa* L., antioxidant, antihypertensive, enzymatic hydrolysis, gummy, hydrolysed ingredient

## Abstract

This study aimed to develop a nutraceutical gummy candy enriched with hydrolysed hemp (*Cannabis sativa* L.) as a natural antioxidant and antihypertensive ingredient. Three European cultivars—Futura 75, Henola, and KC Zuzana—were cultivated under rainfed (RF) and irrigated (RFCI) conditions and assessed for nutritional composition and bioactivity. Henola variety showed the most favourable profile, showing the highest protein content under RFCI (29.4 g 100 g^−1^ d.m.) and the greatest phenolic concentration under RF (15.8 µmol GAE g^−1^ d.m.), with 35–40% higher antioxidant capacity than the other cultivars. Henola (RF) was selected for enzymatic hydrolysis with Ultraflo^®^ XL, which enhanced total phenolics and antioxidant capacity by 65% and 58%, respectively, stabilizing after 18 h. Incorporation of the hydrolysate (0.66%) into a pectin-based gummy significantly (*p* < 0.05) increased total phenolic content by 52% and antioxidant capacity by up to 60% compared with controls. After simulated digestion, bioactivity decreased by 30–45% but remained higher than controls. The incorporation of 0. 66 g of hydrolysed ingredient in 100 g of gummy increased ACE inhibition by 10% after digestion, probably associated with the peptides released during the digestion, confirming hydrolysed hemp as a stable multifunctional ingredient for plant-based nutraceutical formulations targeting oxidative stress and hypertension.

## 1. Introduction

Traditional gummy candies, often rich in added sugars and low in nutritional value, have long been associated with health concerns such as obesity, dental caries, and poor dietary habits—particularly among children and older adults [1]. However, recent innovations in food science and technology are attempting to shift consumer perception of these products by promoting the transformation of conventional gummy candies into natural and functional alternatives through the incorporation of bioactive ingredients such as vitamins, minerals, antioxidants, polyunsaturated fatty acids, probiotics, and prebiotics [2,3].

Among the main strengths of these products are their potential nutritional and bioactive benefits and attractive mode of consumption. For populations with reduced appetite or difficulty swallowing, these gummies offer a convenient and palatable alternative. In children, fortified gummies are used to support immune function and cognitive development, while in older adults, they may contribute to maintaining bone density and joint mobility, preventing nutrient deficiencies, and supporting cardiovascular health. The food industry is also advancing toward formulations that are low in sugar or sugar-free, using plant extracts not only for flavour enhancement but also to provide antioxidant support, immune modulation, improved oral health, glycaemic control, and digestive balance [4]. By preserving both the bioactivity of the incorporated functional ingredients and overall sensory appeal, functional gummies are emerging as a valuable addition to paediatric and geriatric nutritional and pharmaceutical strategies [2].

One particularly promising ingredient in the development of functional foods is hemp (*Cannabis sativa* L.), which is attracting increasing interest due to its robust nutritional and bioactive profile. Hemp-based products offer a sustainable, plant-derived source of high-quality protein, essential fatty acids, dietary fibre, antioxidants, and bioactive peptides—positioning them as health-promoting alternatives to conventional food ingredients [5,6].

The nutritional composition of hemp seeds is significantly influenced by factors such as genotype, soil quality, climate, and agronomic practices [6,7]. Within the European Union (EU), only certified hemp cultivars with low tetrahydrocannabinol (THC) content (<0.2–0.3%)—as listed in the Common Catalogue of Varieties of Agricultural Plant Species—are authorized for cultivation. Cultivars such as Finola and Carmagnola are widely grown for food purposes due to their consistent yield, regulatory compliance, and favourable nutritional profiles [8].

Agronomic conditions also play an important role in phytochemical composition of hemp. For example, irrigation has been shown to increase the cannabinoid content of the inflorescences. In contrast, drought stress promotes the accumulation of metabolites such as amino acids (e.g., alanine and valine), sugars, and organic acids [8]. Moreover, genetic variation among cultivars affects the bioavailability of key minerals. While calcium and iron exhibit low bioaccessibility across varieties, zinc availability varies significantly, with certain cultivars demonstrating enhanced mineral uptake [9].

Within the European Union (EU), hemp seeds and their derivatives—such as dehulled seeds, oils, flours, and protein powders—are considered traditional foods, always from approved low-THC cultivars. However, cannabinoid-rich extracts like cannabidiol (CBD) are classified as novel foods under Regulation (EU) No 2015/2283 [10] and therefore require authorization following safety evaluation by the European Food Safety Authority (EFSA). This regulatory framework ensures food safety while supporting the standardized commercialization of hemp-based products across the EU.

Nutritionally, hemp seeds stand out as a complete protein source, containing all nine essential amino acids. They also offer an optimal omega-6 to omega-3 fatty acid ratio of approximately 3:1 and are rich in vitamins such as vitamin E and B-complex, as well as minerals like magnesium, iron, and zinc [5,6]. Their high dietary fibre content further enhances their nutritional appeal. Importantly, the protein in hemp is both highly digestible and hypoallergenic, making it an excellent choice for vegan and gluten-free dietary regimens.

From a technological point of view, hemp-derived flours and proteins improve water retention, texture, and shelf-life in baked and pasta products. They also function as natural emulsifiers, supporting the development of clean-label and allergen-free formulations [11,12].

Enzymatic hydrolysis of hemp seed proteins has been demonstrated as an effective strategy to enhance their nutritional and functional properties. This process breaks down larger proteins into smaller peptides—typically less than 2 kDa—which are more easily absorbed in the gastrointestinal tract. These peptides offer absorption efficiency comparable to whey protein but without the associated allergenic risks or digestive discomfort. In vitro studies have reported digestibility levels as high as 98%, making hemp protein hydrolysates especially beneficial for individuals with sensitive digestion or elevated protein requirements [13,14].

Beyond enhanced digestibility, these hydrolysates exhibit a variety of bioactivities. They have demonstrated strong antioxidant activity in assays such as DPPH^●^, ABTS^●+^, and FRAP, particularly when produced using enzymes like Alcalase or Flavourzyme under optimized conditions [15]. Alcalase and flavourzyme are proteolytic enzymes commonly applied to seeds to enhance the release and bioaccessibility of bioactive compounds that are naturally bound to their fibre-rich cell walls. Alcalase, a broad-acting endoprotease, helps break down the protein–fibre complexes and structural barriers within seed tissues, freeing phenolics, peptides, and other beneficial compounds trapped in insoluble fibre. Meanwhile, flavourzyme, which combines endo- and exopeptidase activities, further degrades residual proteins and produces smaller, more soluble peptides that promote the liberation of fibre-associated bioactives. When used individually or in combination, these enzymes disrupt seed cell wall integrity and reduce molecular entrapment, ultimately increasing the accessibility and potential bioavailability of polyphenols, antioxidants, and other health-promoting compounds originally confined within the seed fibre matrix. The action of enzymes can directly or indirectly release hemp-derived peptides that show angiotensin-converting enzyme (ACE) inhibitory activity, which is relevant to blood pressure management [16]. A notable double-blind crossover study with hypertensive adults reported that daily intake of 5 g of hemp protein hydrolysate combined with 45 g of hemp protein isolate lowered significantly both systolic and diastolic blood pressure compared to a control group consuming casein [16].

Also, these aspects have been studied using proteomic tools showing that these peptides have multi-target biological effects and inhibit enzymes such as dipeptidyl peptidase-IV (DPP-IV) and renin, which are involved in type 2 diabetes and hypertension, respectively. Moreover, they stimulate glucose uptake and modulate immune responses, suggesting a broad therapeutic potential in metabolic and inflammatory conditions [17]. These bioactivities are largely attributed to hydrophobic amino acid residues that enhance peptide stability and facilitate cellular uptake.

In this context, the present study aimed to develop functional gummy candy enriched with hemp seed protein hydrolysates. To achieve this objective, four phases were planned. In the first phase, genetic and agronomic aspects were evaluated. Three EU-authorized hemp cultivars—Futura 75, Henola, and KC Zuzana [18]—were studied and cultivated under two different agronomic conditions (rainfed vs. rainfed and irrigation) in terms of their nutritional and bioactive (antioxidant and antihypertensive) properties. In the second phase, a biotechnological process based on a hydrolytic procedure was optimized in order to maximize the bioavailability of active compounds. At the end of this process, a hydrolysed ingredient will be produced and stabilized. The third phase was based on the formulation of the enriched gummy candy based on its textural and organoleptic properties, and in the last phase, a simulated dynamic digestion was applied to evaluate an in vitro approximation of a real-world scenario. These findings aimed to lay the groundwork for future clinical studies validating the efficacy of hemp-based functional gummies in supporting metabolic health.

## 2. Materials and Methods

### 2.1. Chemicals

Sodium carbonate decahydrate, Folin–Ciocalteu (FC) reagent, iron (III) chloride hexahydrate, fluorescein, 2,2-diphenyl-1-picrylhydrazyl (DPPH^●^), gallic acid (GA), iron (II) sulphate heptahydrate, cellulose, 2,2′-azino-bis (3-ethylbenzothiazoline-6-sulfonic acid) diammonium salt (ABTS^●+^), 2,2′-azobis(2-methylpropionamidine) dihydrochloride (AAPH), 2,4,6-tri(2-pyridyl)-s-triazine (TPTZ), *o*-aminobenzoylglycyl-*p*-nitro-L-phenylalanyl-L-proline (Abz-Gly-Phe(NO_2_)-Pro), *o*-aminobenzoylglycine (Abz-Gly), 6-hydroxy-2,5,7,8-tetramethyl-2-carboxylic acid (Trolox), sodium carboxymethylcellulose (CMC), and angiotensin I-converting enzyme (ACE) were supplied from Sigma-Aldrich, Co. (St. Louis, MO, USA). Hydrochloric acid, glacial acetic acid, formic acid, and sodium acetate were purchased from PanReac AppliChem (ITW Reagents, Darmstadt, Germany). Novozymes (Bagsværd, Copenhagen, Denmark) kindly supplied UltraFlo^®^ XL food-grade enzymes.

### 2.2. Raw Material

The varieties were cultivated in experimental fields of the Agricultural Technological Institute of Castilla y León (ITACyL, Valladolid, Spain) under control agronomic practise. The varieties were cultivated using rainfed conditions (RF, 70 L m^−2^ of rainfall) and rainfall supplemented with conventional irrigation (RFCI, 350 and 362 L m^−2^) throughout the entire cycle. The meteorological information is registered in the InfoRiego platform in our institution: https://www.inforiego.org/opencms/opencms/) (accessed on 23 November 2025). Crops were harvested when they reached approximately 70% maturity. Once collected, the seeds were dried at 40 °C until their moisture content decreased to approximately 10–7%, and then maintained in the dark at room temperature until they were processed. The three varieties had different flowering cycles and origins. On one hand, the Henola variety, of Polish origin, has an early flowering cycle, KC Zuzana is a Hungarian variety with an intermediate cycle, and finally, the French variety Futura 75 has an intermediate–late cycle. Three varieties are commercial, and all are registered on CPVO (Community Plant Variety Office) https://cpvo.europa.eu/en (accessed on 23 November 2025). Additionally, the references for all of them are Kc-Zuzana: 363053, Futura 75: 213947, and Henola: 213958.

**Figure 1:**
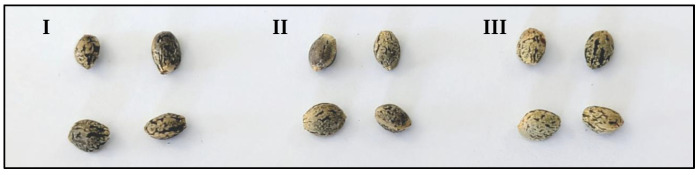
*Cannabis sativa* L. varieties used for the screening. Henola (**I**), KC Zuzana (**II**), and Future 75 (**III**).

Also, the varieties were selected based on their differing compositional characteristics. Specifically, the variety *Futura 75* is known for its high protein content, *KC Zuzana* for its high fat content, and *Henola* for its high fibre content (Figure 1). All samples were properly labelled, vacuum-sealed, and stored at the facilities of ITACyL until processing.

### 2.3. Processing Route

**Figure 2:**
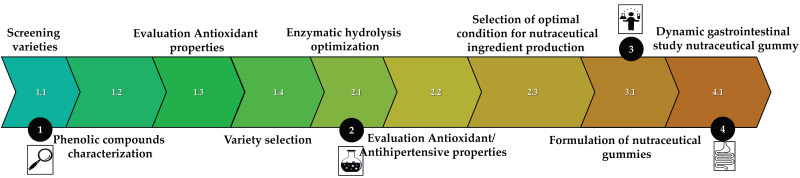
Processing route for the development of antioxidant and antihypertensive gummy candy.

The flowchart outlines (Figure 2) the process of developing nutraceutical gummy candy. The process started with screening of three food hemp authorized seed varieties grown in the ITACyL agronomic fields under feed irrigation or hydric stress. The screening was focussed on their phenolic content (TPC) and total antioxidant capacity (TAC).

The variety with the highest total phenolic content (TPC) and antioxidant capacity (TAC) was subjected to a hydrolytic process to enhance the bioavailability of its phenolic compounds. This process was optimized based on TPC, TAC, and antihypertensive activity. The resulting extract, produced under the optimized hydrolysis conditions, was stabilized through freeze-drying and incorporated into a gummy formulation. To further evaluate the efficacy of the extract within the gummy matrix, the final product underwent simulated human gastrointestinal digestion. Following digestion, the gummy samples were analysed for total phenolic content (TPC), total antioxidant capacity (TAC), and angiotensin-converting enzyme (ACE) activity to better approximate in vivo conditions.

### 2.4. Nutritional and Bioactive Characterization of Hemp (Cannabis sativa *L.*) Seed Varieties

All hemp seed varieties stored at room temperature with 7–10% humidity were manually cleaned and ground using a mill (Model Cyclotec 1093, Foss, Hilleroed, Denmark) equipped with a 0.5 mm sieve. Immediately afterward, the material was vacuum-packed in plastic bags and stored at −20 °C until analysis.

#### 2.4.1. Nutritional Composition

The nutritional composition of the hemp seeds was assessed for all tested varieties and agronomic conditions. Total protein content was quantified using the Dumas method (990.03) [19] with an elemental analyser (LECO Corp., St. Joseph, MI, USA). A conversion factor of 5.37 [20] was applied to obtain the protein values. Total fat was measured through petroleum ether extraction (40–60 °C) for 4 h using a Soxhlet apparatus (AOAC 2005, method 2003.05) [19]. Moisture was determined by drying three grams of ground sample (WB, OH) at 105 °C for 3 h. Ash content was obtained by incinerating the material at 550 °C for 5 h in a muffle furnace (AOAC 2005, method 923.03) [19]. Carbohydrate content was calculated by difference. Total dietary fibre (TDF) was measured following AOAC method 985.29 [19], employing the TDF100A-1KT kit from Sigma (St. Louis, MO, USA). Results were reported as grams per 100 g of dry matter (d.m.). All determinations were carried out in duplicate.

#### 2.4.2. Determination of Total Phenolic Content (TPC)

Total phenolic content was evaluated in seed hemp free and bound phenolic extracts, hydrolysed extracts, and digested gummies.

Contents of free phenolic compounds were measured in methanolic extracts prepared from the seeds. In summary, 1 g of sample was mixed with 20 mL of a methanol–water solution (1:1, *v*/*v*; adjusted to pH 2 with 0.1 M HCl) and agitated in an orbital shaker (250 rpm, 25 °C, 1 h). After centrifugation (25 °C, 3800× *g*, 10 min), the resulting supernatant was collected and reextracted after the sample was filtered. Finally, the extract was evaporated and reconstituted in 10 mL of methanol: water (1:1, *v*/*v*; acidified to pH 2 with 0.1 M HCl). Samples were filtered through a 0.22 µm membrane and subsequently stored at −80 °C.

For bound phenolic compounds, the residues after extraction of free phenols were processed through a two-step hydrolysis—first under alkaline conditions (10 M NaOH) and then under acidic conditions (pH 2, HCl)—to release the bound (insoluble) phenolic fraction, following the procedure described by Mattila et al. [21] and adjusted by Rico et al. [22] for 16 h. The solution was then acidified to pH 2, and the released phenolic compounds were extracted three consecutive times using 15 mL of ethyl acetate, with manual shaking followed by centrifugation. Once the alkaline hydrolysis step was finished, an additional acidic hydrolysis was carried out by adding 2.5 mL of concentrated HCl to the tube and incubating it in a water bath at 85 °C for 30 min. After cooling, the ethyl acetate extraction was repeated following the same procedure used after the alkaline step. The organic phases were pooled, evaporated to dryness, and reconstituted in 10 mL of methanol. The extracts containing bound phenolics were filtered through a 0.22 µm membrane and stored at −80 °C until analysis.

Free and bound phenolic extracts were qualitatively assessed using the Folin–Ciocalteu assay [23], employing gallic acid (GA) as the calibration standard. All measurements were performed in duplicate. The final values were adjusted according to the moisture content and expressed as µmol GA equivalents (GA Eq) g^−1^ d.m.

#### 2.4.3. Determination of Total Antioxidant Capacity (TAC)

Total antioxidant capacity (TAC) of hemp seed free and bound phenolic extracts, hydrolysed fractions, and digested gummies was evaluated using the following assays: the DPPH^●^ (2,2-diphenyl-1-picrylhydrazyl) radical method, the ABTS^●+^ (2,2′-azinobis-(3-ethylbenzothiazoline-6-sulfonate) assay, the oxygen radical absorbance capacity (ORAC) test, and the ferric reducing antioxidant power (FRAP) method.

The DPPH^●^ assay was performed following the method of Brand-Williams et al. [24] with slight modifications. Sample extract (250 µL), Milli-Q water, and a 120 µM DPPH^●^ solution in methanol were combined in a 1:4:5 ratio (*v*:*v*:*v*). After 30 min, absorbance was recorded at 525 nm using a Spectrostar Omega microplate reader (BMG Labtech, Ortenberg, Germany). A Trolox standard curve (7.5–210 µM) was used to quantify DPPH^●^ scavenging activity, and results were expressed as micromoles TE per 100 g d.m.

The ABTS^●+^ assay was conducted following the method described by Re et al. [25], as modified by Martin-Diana et al. [26]. To prepare the stock solution, 7 mM ABTS^●+^ was combined with 2.45 mM potassium persulfate in a 1:1 (*v*:*v*) ratio and allowed to react overnight at room temperature in the dark. The working solution was obtained by diluting the stock solution with 75 mM phosphate buffer (pH 7.4) until reaching an absorbance of 0.7 ± 0.02 at 730 nm, followed by equilibration at 30 °C. Then, 20 µL of sample extract, blank, or Trolox standard (7.5–210 µM) was mixed with 200 µL of the ABTS^●+^ working solution in a 96-well microplate. After incubating for 1 h in darkness, absorbance was read at 730 nm using a Spectrostar Omega microplate reader (BMG Labtech, Ortenberg, Germany). Results were expressed as micromoles TE per 100 g d.m.

The ORAC assay was performed according to a method previously reported by Ou et al. [27], with some adaptations. The Trolox calibration curve (7.5–210 µM) and the samples were prepared in 75 mM phosphate buffer (pH 7.4). Fluorescence was monitored for 150 min at 37 °C using a CLARIOstar Plus microplate reader (BMG, Ortenberg, Germany) with excitation at 485 nm and emission at 520 nm. The antioxidant capacity was calculated from the area under the fluorescein decay curves obtained for the samples compared with the blank, and results were expressed as micromoles TE per 100 g d.m.

The FRAP assay was carried out following the procedure of Benzie and Strain [28]. The FRAP working reagent was prepared by combining 300 mM acetate buffer (pH 3.6), 10 mM TPTZ dissolved in 40 mM hydrochloric acid, and 20 mM ferric chloride hexahydrate in a 10:1:1 (*v*:*v*:*v*) ratio. For the reaction, 20 µL of the sample, blank, or standard was mixed with 1.9 mL of the freshly prepared FRAP solution. Distilled water served as the blank, while ferrous sulphate heptahydrate (400–3000 µM) was used to build the calibration curve. Absorbance was recorded at 593 nm using a Spectrostar Omega microplate reader (BMG Labtech, Ortenberg, Germany). Results were expressed as micromoles of Fe^+2^ equivalents per 100 g of d.m.

### 2.5. Optimization of Enzymatic Hydrolysis of Hemp Seed (Cannabis sativa *L.*)

To evaluate enzymatic activity, Ultraflo^®^ XL enzyme was used based on the efficacy enhancing bioaccessibility of phenolic compounds in previous studies by the authors [29,30]. The process involved grinding of hemp seeds and mixing with 40 mL of distilled water (1:20 *w*:*v*), adjusting the pH to 5 with 1.5 M malic acid. A 1% enzyme-to-solid ratio was used, and samples were incubated at 47 °C in a thermostatic bath with agitation. This environment promotes the enzymatic breakdown of structural polysaccharides, enabling the release of intracellular phenolic compounds and lignans, typically trapped within vacuoles or bound to the cell wall. Hydrolysis was monitorized at 0, 1, 2, 4, 6, 18, 20, and 24 h to generate a complete kinetic profile. At each time point, 1 mL of hydrolysate was sampled, heat-inactivated (95 °C, 10 min), centrifuged at 13,500 rpm for 3 min, and supernatant stored at −20 °C. Following hydrolysis, the samples were lyophilized using a Telstar LyoBeta unit Terrassa (Barcelona, Spain). The freeze-drying program consisted of 30 h of primary drying and 8 h of secondary drying, following these steps: freezing at −40 °C for 2 h, after which the condenser was prepared, and vacuum was applied to the chamber. Primary drying proceeded at −20 °C for 6 h under a pressure of 200 µbar, followed by sequential stages at −10 °C, 0 °C, 10 °C, and 20 °C, each maintained for 6 h at the same pressure. The procedure was completed with an 8 h secondary drying phase at 20 °C, finalizing the entire cycle. Dried samples were stored for further analysis.

### 2.6. Angiotensin-Converting Enzyme (ACE)-Inhibition Activity

The ACE-inhibitory activity was evaluated following the procedure described by Shalaby et al. [31], with some modifications [32] for hydrolysed hemp extracts and gummies digested using a dynamic system. ACE from rabbit lung (purchased from Sigma Aldrich 2 U) was reconstituted in 8 mL of a mixture of 50 mM tris-HCl buffer (with 2 μM ZnCl_2_, pH 7.5) and glycerol (1:1, *v*:*v*) to produce ACE stock solution (250 mU/mL). Aliquots were made and stored at −20 °C. Each day of the assay, the ACE working solution (25 mU/mL) was prepared by diluting the stock solution 1:9. The FAPGG solution was prepared by dissolving 5.991 mg with 10 mL of 50 mM tris-HCl buffer (with 0.3 M NaCl, pH 7.5). The samples employed were the same as used for the TAC determinations, making different dilutions (1, 2, 5, 10, 20, 30) in the extraction solvent to test different concentrations. The reaction was performed by adding 50 μL of milli-Q water (as blank) or sample and 90 μL of ACE working solution in a black 96-well microplate and incubating at 37 °C for 5 min. Then, 135 μL of the FAPGG solution was added, except to the blank, and the absorbance was measured at 340 nm for 85 min at 37 °C. Captopril was included as a reference ACE inhibitor. The reaction slopes were used to determine ACE activity, and the percentage (%) of inhibition was calculated. The concentration required to inhibit 50% of the enzyme activity (IC_50_) was determined by plotting the logarithm of the sample concentration (log) against the ACE % inhibition.

### 2.7. Formulation of Gummy Candy Enriched with Hemp (Cannabis sativa *L.*) Hydrolysed Ingredient

Pectin, sucrose, lemon juice, and ginger were used to develop the emulgel for the preparation of candy. The control gummy (CG) was prepared using structuring agent (pectin solution) hydrated at 60 °C with continuous stirring using a temperature-controlled magnetic stirrer. Sucrose was mixed with lemon juice as a low molecular co-solute which acted as a dehydrating agent to initiate gelation. After water was added to the mixture, it was heated to approximately 90–95 °C. After agave syrup was added, the mixture was stirred to ensure homogenization of all the ingredients (Table 1).

The final mixture was brought to a gentle boil (approximately 100 °C, 3 min). Ginger was added in the mixture, and it was allowed to cool down to around 50–60 °C, then poured into molds to shape the gummies. In the case of EG, the hydrolysed ingredient was added to the formula when temperature was lowered to 50 °C to avoid the loss of activity of thermolabile bioactive compounds. The concentration of hydrolysed ingredient (0.66%) was selected based on commercial gummies enriched with bioactive compounds as reference. The molds were placed in a freezer at −20 °C for 2 h to set the gummies firmly. After setting, the gummies were removed and dried at room temperature (around 20–25 °C) for 24 h to remove excess moisture. Once dried, gummies were cut into four equal pieces (2 g) and coated with chitosan solution (1:4 *w*:*w*), then dried again for 1 h at room temperature (RT). Finally, the gummies were stored at refrigeration temperature (4 °C) until further analysis.

### 2.8. Dynamic Digestion Model of Gummy Candies Fortified with Hemp (Cannabis sativa *L.*) Hydrolysed Ingredient

The control and enhanced gummy candies were digested in vitro following the standardized method for simulating human gastrointestinal digestion in vitro, INFOGEST, adapted to the ITACyL dynamic digestion prototype [33,34]. The equipment was prepared with the required solutions for pH control (1 M HCl and 0.5 M NaHCO_3_), and each compartment—stomach, duodenum, jejunum, and ileum—was filled with its respective simulated medium (gastric or intestinal), keeping the stomach chamber at half capacity. Each section consisted of a plastic vessel placed inside a cylinder through which warm water circulated to maintain a constant temperature of 37 °C. The working volumes were 100 mL for the stomach and a total of 100 mL for the duodenum, jejunum, and ileum combined. Six grams of sample were weighed, mixed with 40 mL of distilled water and 6 mL of simulated salivary fluid, homogenised, and incubated at 37 °C for 2 min. The mixture was then transferred into the stomach compartment to begin the digestion protocol. Flow rates were set to 1 mL/min during the gastric stage and 2 mL/min during the intestinal stage. Continuous pH regulation was carried out in each phase using pH meters and automated dosing pumps, keeping the gastric phase at pH 3 and the intestinal phase at pH 7. Sample collection was carried out by collecting the effluent at the system outlet from minute 120 to minute 360. Finally, enzymatic activity was stopped by heating the samples to 95 °C for 10 min.

Finally, all digested samples were freeze-dried using a Telstar LyoBeta unit. The freeze-drying cycle included 30 h of primary drying and 8 h of secondary drying. The procedure began with a freezing step at −40 °C for 2 h, followed by condenser conditioning and the application of vacuum to the chamber. Primary drying was then carried out at −20 °C for 6 h at 200 µbar, after which the temperature was gradually increased to −10 °C, 0 °C, 10 °C, and 20 °C, maintaining each stage for 6 h at the same pressure. The cycle was completed with secondary drying at 20 °C for 8 h.

### 2.9. Texture Analysis of Gummies Fortified with Hemp (Cannabis sativa *L.*) Ingredient

The firmness was evaluated as the force required to produce a deformation of 4 mm at a speed of 4 mm s^−1^ using a texture analyser (ANAME TA.XTPLUS, Stable Micro Systems LTD., Vienna Court, UK) equipped with a flat-faced cylindrical probe of 4 mm in diameter. Gummy candies had a dimension of 10 × 10 × 10 mm and were evaluated in duplicate.

### 2.10. Sensory Analysis of Gummies Fortified with Hemp (Cannabis sativa *L.*) Ingredient

The sensory analysis was conducted by an internal panel of ITACyL staff aged between 23 and 64, with prior experience in sensory evaluation of different food products. A descriptive analysis was performed, assessing the intensity of defined visual parameters such as colour, appearance (transparency), and overall acceptability. Aroma was evaluated for citrus and smoky; for flavour, sweetness, sourness, bitterness, and smoke attributes were assessed for all the gummy candies. Texture was evaluated through both finger and mouth assessments—elasticity and hardness were measured with the fingers, while cohesiveness and hardness were evaluated in the mouth. All parameters were rated on a 1–9 scale, where 1 represented “low”, 5 indicated “moderate”, and 9 signified “extreme” for each attribute.

### 2.11. Statistical Analysis

The results were reported as mean ± standard deviation from a minimum of three independent experiments. To identify statistically significant differences among the means, an analysis of variance (ANOVA) followed by Duncan’s post hoc test was applied. All statistical procedures were carried out using Statgraphics Centurion XVI^®^ (StatPoint Technologies, Inc., Warrenton, VA, USA).

## 3. Results and Discussion

### 3.1. Proximate Composition of Hemp Cultivars Under Hydric Stress and Conventional Practice

The proximate composition of three hemp seed (*Cannabis sativa* L.) varieties (Futura 75, Henola, and KC Zuzana) grown under rainfall (RF) and rainfall supplemented with conventional irrigation (RFCI) was assessed. Significant (*p* ≤ 0.05) differences were observed in ash and macronutrient (fat, total dietary fibre, protein, and carbohydrates) content, which were influenced by both genotype and water availability (Table 2), in agreement with the literature where many authors have reported differences in nutritional composition due to genotype and environmental growth factors in hemp seeds. Leonard et al. [35], Farinon et al. [36], and Callaway [6] showed that these seeds typically contain 94% dry matter, 20–25% protein, 25–35% lipids, 20–30% carbohydrates (mostly dietary fibre), and 5–6% ash, in agreement with the profiles of the varieties tested similarly and the results observed previously by the authors.

Looking at individual macronutrients, results showed that ash levels varied significantly (*p* ≤ 0.05) among cultivars, reflecting a direct difference in mineral accumulation capacity. The results were consistent with those obtained by other authors that studied hemp seed germplasm [37]. These findings highlight that mineral content is not only affected by soil and irrigation conditions but is also strongly genotype-dependent. Values were similar to values reported by other studies of Futura 75 and KC Zuzana [37,38].

Water availability significantly (*p* ≤ 0.05) affected protein content, yielding increases of 12–15%. Among cultivars, Henola and KC Zuzana had higher levels than Futura 75, corroborating studies that identified Henola as a protein-rich cultivar, due to genetic selection for seed productivity and nutritional quality [39]. KC Zuzana also exhibited elevated protein content, suggesting that central European cultivars may serve as promising candidates for protein-enriched food applications. The observed protein increases under irrigation were consistent with patterns reported in oilseeds, where improved water status enhances amino acid assimilation and storage protein deposition [40].

Fat content was highest in KC Zuzana across both cultivation conditions, followed by Henola, whereas Futura_75 consistently exhibited the lowest fat levels, particularly under water-limited conditions. Irrigation increased fat content especially in KC Zuzana, supporting the hypothesis that improved water availability stimulates lipid biosynthesis. This aligns with findings in hemp and other oilseeds where irrigation promoted lipid accumulation at the expense of carbohydrate reserves [40]. However, the extent of this effect differed among the varieties, underlining the importance of genotype and water availability.

Carbohydrate content had an inverse relationship with protein and fat accumulation. Futura 75 consistently showed the highest carbohydrate levels, confirming previous observations that cultivars with limited oil biosynthesis tend to accumulate carbohydrates [6]. Such results are particularly valuable for carbohydrate-based nutraceuticals and functional food applications.

The TDF fraction exhibited a complex, genotype-dependent response. While Futura 75 maintained consistently higher TDF levels under both cultivation regimes, some varieties showed reduced fibre under irrigation. This trend suggests that increased water availability may promote lipid and protein storage at the expense of structural carbohydrates. The resilience of Futura 75 in maintaining high TDF and carbohydrate content under RF positions it as a promising cultivar for fibre- and carbohydrate-oriented applications, including functional foods with prebiotic potential [37].

Protein content was significantly (*p* ≤ 0.05) higher in Henola and KC Zuzana compared to Futura 75, regardless of irrigation, which is consistent with previous findings reporting elevated protein levels in Henola due to its genetic selection for seed yield and nutritional quality [40]. Carbohydrate content followed an inverse trend, with higher levels in Futura 75, corroborating earlier observations that cultivars with lower oil deposition tend to accumulate more carbohydrates [6].

Results showed that hemp seed composition was strongly modulated by both genetic aspects and water availability, though their effects vary among macronutrients. Irrigation generally enhanced protein and lipid content, with KC Zuzana showing the strongest response. Conversely, Futura 75 consistently retained higher carbohydrate and fibre contents, underscoring its potential for applications targeting carbohydrate or dietary fibre enrichment. These findings emphasize that both breeding and agronomic practices should consider not only yield but also nutritional quality, tailoring strategies to the intended end-use of the crop (protein-, oil-, or fibre-oriented).

The amount of water available during the growth seems to be as important to the proximal composition as the genetic component of the cultivar. Futura 75 was the variety with higher TDF and total carbohydrate content in RF, and KC Zuzana, regardless of the irrigation method, reached the highest values for fats and protein. These results agreed with those of other authors [41], highlighting the variability in oil accumulation among hemp cultivars and water availability. TDF showed different patterns; some varieties cultivated under dry conditions showed higher fibre concentrations, whereas others presented reductions when exposed to irrigation. This suggests a genotype-dependent response, where certain varieties were more resilient in preserving structural carbohydrate fractions under variable water availability.

### 3.2. Total Phenolic Content (TPC) in Free and Bound Fractions Under Irrigation and Dry Conditions

**Figure 3:**
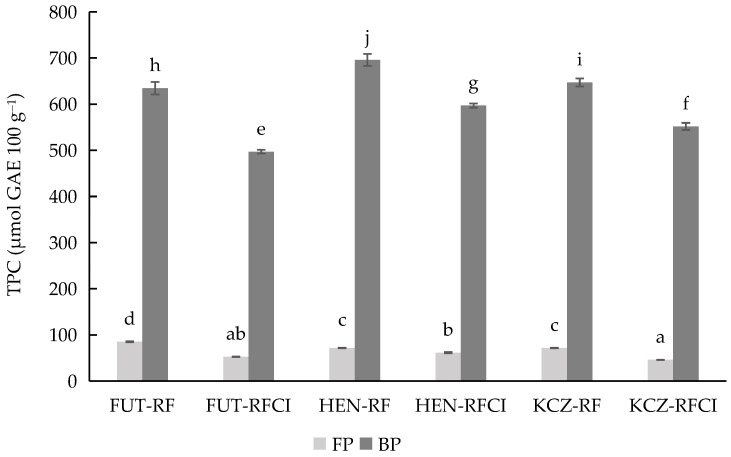
Total phenolic content (TPC) for free phenolic fraction (FP) and bound phenolic fraction (BP) of three *Cannabis sativa* L. varieties: Futura 75, Henola, and KC Zuzana, cultivated under rainfall (RF) and rainfall and conventional irrigation conditions (RFCI). Results were expressed in micromoles GAE 100 g^−1^ of dry matter. Abbreviations: TPC, Total Phenol Content; GAE, Gallic Acid Equivalents; FUT, Futura 75 variety; HEN, Henola variety; KCZ, KC Zuzana variety; RF, under rainfall; RFCI, rainfall and conventional irrigation conditions. Different lowercase letters indicate significant differences in the mean values (one-way ANOVA, Duncan’s test, *p* ≤ 0.05) between samples.

The analysis of total phenolic content (TPC) showed significant (*p* ≤ 0.05) variation among *Cannabis sativa* L. varieties, water availability, and between free (FP) and bound phenolic (BP) fractions (Figure 3). Across all cultivars, the bound phenolic fraction was higher than the free phenolic fraction regardless of water availability, with Henola being the variety with higher total phenolic content, followed by Futura 75 and KC Zuzana. RFCI reduced phenolic accumulation in all varieties, particularly in bound phenolic fractions, although the effect was not uniform across all genotypes. This suggests that water availability modulates phenolic biosynthesis differently depending on the genetic aspects of each cultivar.

These patterns were consistent with other studies reported by other authors, indicating that hemp seeds are rich in phenolic compounds, with substantial variability driven by genotype and environment [42,43]. The reduction in phenolics under irrigation may reflect a lower stress response, since drought and environmental limitations are known to trigger enhanced phenolic biosynthesis as part of plant defence mechanisms [44]. Therefore, the high TPC in RF Henola suggests it may be a superior source of bioactive compounds for nutraceutical development.

### 3.3. Total Antioxidant Capacity (TAC) in Free and Bound Fractions Under Rainfall (RF) and Rainfall and Conventional Irrigation Conditions (RFCI)

**Figure 4:**
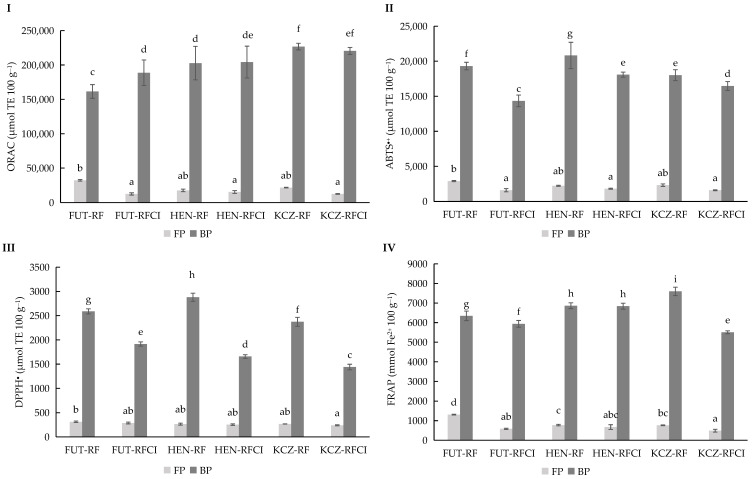
ORAC (**I**), ABTS^●+^ (**II**), DPPH^●^ (**III**), and FRAP (**IV**) values for free phenolic fraction (FP) and bound phenolic fraction (BP) of three *Cannabis sativa* L. varieties: Futura 75, Henola, and KC Zuzana, cultivated under rainfall (RF) and rainfall and conventional irrigation conditions (RFCI). Results were expressed in micromoles TE 100 g^−1^ of dry matter for ORAC, ABTS^●+^, and DPPH^●^ and in millimoles Fe^2+^ 100 g^−1^ for FRAP. Abbreviations: TE, Trolox Equivalents; FUT, Futura 75 variety; HEN, Henola variety; KCZ, KC Zuzana variety; RF, under rainfall; RFCI, rainfall and conventional irrigation conditions. Different lowercase letters indicate significant differences in the mean values (one-way ANOVA, Duncan’s test, *p* ≤ 0.05) between samples.

The antioxidant evaluation using the four complementary assays (ABTS^●+^, DPPH^●^, FRAP, and ORAC) showed marked and significant (*p* ≤ 0.05) differences across varieties and between free and bound phenolic fractions (Figure 4). Bound fractions consistently exhibited higher antioxidant activity than free fractions, regardless of the parameter evaluated. Among the varieties, Henola had the strongest antioxidant potential across most assays, followed by KC Zuzana and Futura 75, which presented comparatively lower activity. Irrigation reduced antioxidant capacity in several cases, particularly in free phenolic fractions, indicating that phenolic-mediated antioxidant responses are enhanced under stress conditions such as limited water availability, correlating with the results for TPC content (Figure 3).

The antioxidant activity of hemp (*Cannabis sativa* L.) was highly variable and depends on both the seed variety and water availability. Studies using multiple antioxidant assays (such as ABTS^●+^, DPPH^●^, FRAP, and ORAC) have shown that bound phenolic fractions generally exhibited higher antioxidant activity than free fractions, regardless of the parameter evaluated (Figure 4). Among the varieties tested, Henola showed the strongest antioxidant potential, which is closely linked to its higher total phenolic content (TPC) compared to KC Zuzana and Future 75 [40,45,46]. Limited irrigation or drought stress tends to enhance antioxidant capacity, especially in the bound phenolic fraction, by stimulating the synthesis of secondary metabolites such as phenolics [47], which was consistent with findings in other oilseeds, where stress conditions promote the accumulation of bioactive compounds with antioxidant properties.

The results confirmed that selecting specific hemp varieties and optimizing cultivation strategies—such as managing irrigation—can significantly enhance the antioxidant functionality of hemp, making it a promising candidate for nutraceutical applications. The results showed that Henola cultivated under RF was the best candidate to continue in phase II since it showed the highest values among different markers associated with antioxidant activity or reducing availability.

### 3.4. Optimization of Enzymatic Hydrolysis of Hemp: Effect on Total Phenolic Content

**Figure 5:**
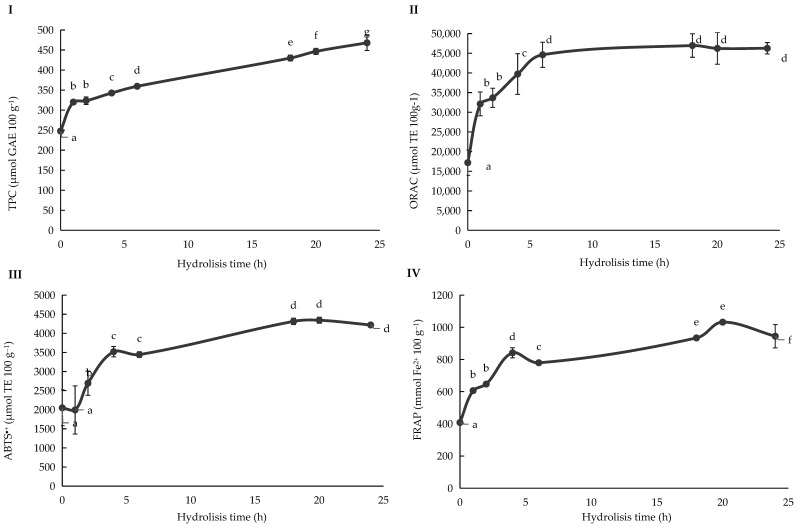
Changes in total phenolic content (TPC) and antioxidant activity (ORAC, ABTS^●+^, FRAP) throughout the enzymatic hydrolysis of *Cannabis sativa* L. Henola seeds grown under rainfed (RF) conditions. Kinetic hydrolytic process TPC (**I**), ORAC (**II**), ABTS^●+^ (**III**), and FRAP (**IV**) values for *Cannabis sativa* L. Henola variety cultivated under rainfall (RF) irrigation conditions. Abbreviations: GAE, Gallic Acid Equivalents; TE, Trolox Equivalents. Different lowercase letters indicate significant differences in the mean values (one-way ANOVA, Duncan’s test, *p* ≤ 0.05).

Following the first phase, Henola under rainfall-fed (RF) conditions was selected as the most suitable variety for subsequent processing, based on its nutritional profile and antioxidant properties (Figure 5). Although Henola is primarily recognized for its high fibre content, it was found to have a competitive protein profile, making it by balance the most favourable candidate for enzymatic hydrolysis. In the second phase, enzymatic hydrolysis was performed, observing a dynamic, time-dependent pattern in total phenolic content (TPC). During the initial stages, a rapid increase in TPC was observed, likely attributable to the solubilization of free phenolic compounds, indicating enzymatic cleavage of ester linkages between phenolic acids (e.g., ferulic and p-coumaric acids) and cell wall polysaccharides. Concurrently, a slower increase in bound phenolic compounds was detected, consistent with their stronger association within the cell wall matrix.

After approximately 18 h, the release of phenolic compounds reached a plateau, suggesting that the majority of accessible phenolics had been liberated during the hydrolytic process.

The role of Ultraflo^®^ XL enzyme is particularly relevant in this context. Several studies have shown that Ultraflo^®^ XL enzyme treatment significantly boosts TPC in hemp matrices by degrading hemicellulose and β-glucan chains, thereby liberating esterified phenolic acids [29]. For instance, Ryu et al. [48] reported that Ultraflo^®^ XL enzyme pretreatment of hemp seed hulls increased free phenolic acids by more than 30%, particularly associated with the bioaccessibility of compounds such as ferulic and p-coumaric acids, which are normally trapped in lignocellulosic networks. Similarly, Wang et al. [49] reported that enzymatic hydrolysis of hemp protein isolates using enzymes enhanced solubilization of phenolics compared to untreated controls. These studies confirm that the enzymatic breakdown of complex polysaccharide–phenolic linkages, either by digestive enzymes or commercial biocatalysts like Ultraflo^®^ XL enzyme, is essential for maximizing phenolic release. Comparing between RF and RFCI, hydrolysates consistently showed higher TPC than undigested samples, with digested hydrolysates (H-D) achieving the highest values. RFCI fractions tended to exhibit greater increases, possibly due to irrigation-induced changes in cell wall hydration, which facilitated subsequent enzymatic breakdown, a trend also noted in genotype × environment studies [40].

### 3.5. Optimization of Enzymatic Hydrolysis of Hemp: Effect on Total Phenolic Content (TPC) and Total Antioxidant Capacity (TAC)

The antioxidant activity of hemp fractions, measured via ABTS^●+^, FRAP, ORAC, and DPPH^●^, reflected the differential release and activity of phenolics and peptides during hydrolysis. ABTS^●+^ scavenging capacity increased rapidly within 5–10 h, consistent with the release of phenolic acids with strong electron-donating ability. Studies using Ultraflo^®^ XL enzyme corroborate this behaviour: Ryu et al. [48] found that ABTS^●+^ scavenging activity of hemp seed hulls increased significantly after Ultraflo^®^ XL enzymatic treatment, reflecting the liberation of caffeic acid and other hydroxycinnamic acids with high radical scavenging efficiency. Reducing power also was analysed using FRAP. Values also increased notably during hydrolysis but stabilized earlier (~10–12 h), indicating that most ferric-reducing phenolics were released in the initial phases. Enzymatic treatments such as Ultraflo^®^ XL similarly enhanced FRAP in hemp-based products [50], demonstrating that enzymatic release of phenolics and bioactive peptides increases the ferric-reducing potential of hemp fractions.

ORAC values displayed a steady increase, reflecting improved capacity to neutralize peroxyl radicals. The sharpest rise occurred between 5 and 10 h, after which values stabilized, suggesting sustained release of chain-breaking antioxidants such as tocopherols, flavonoids, and lignanamides. Lanzoni et al. [51] also emphasized the role of digestion in generating peptides with significant ORAC activity. In hemp, Ultraflo^®^ XL enzyme pretreatment was reported to amplify ORAC values by enhancing the release of soluble antioxidants and potentially synergizing with endogenous peptides [52]. DPPH^●^ activity improved more modestly compared to ABTS^●+^ and FRAP. This reflects the steric hindrance of the DPPH^●^ radical and the lower affinity of certain hemp phenolics for this assay [53]. Lanzoni et al. [51] similarly found DPPH^●^ to be less sensitive to digestion-induced phenolic release. Studies with Ultraflo^®^ XL enzyme confirm this selectivity: while ABTS^●+^ and FRAP increased significantly after the enzymatic treatment, DPPH^●^ responses remained less pronounced, highlighting assay-specific sensitivities to the released antioxidant profile. The results showed that Ultraflo^®^ XL enzyme pretreatment breaks down polysaccharide–phenolic and protein–phenolic bonds, enhancing the solubilization of phenolic compounds. ABTS^●+^ and FRAP were the most responsive parameters, reflecting electron transfer and reducing capacity; ORAC captured sustained peroxyl radical protection; while DPPH^●^ exhibited limited sensitivity to hydrolysis-induced changes.

### 3.6. In Vitro Digestion of Henola Hydrolysate Ingredient

**Figure 6:**
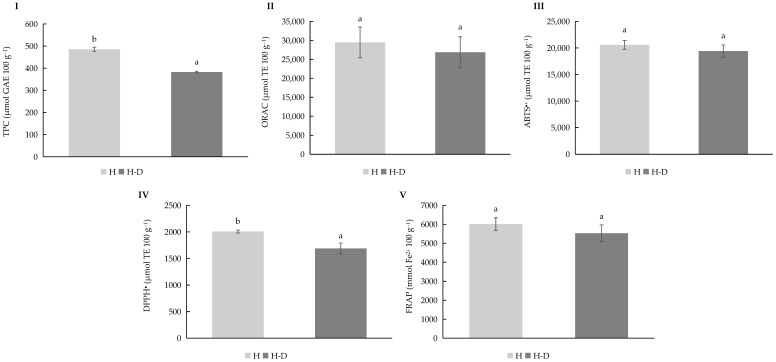
TPC (**I**), ORAC (**II**), ABTS^●+^ (**III**), DPPH^●^ (**IV**), and FRAP (**V**) values for *Cannabis sativa* L. Henola variety cultivated under rainfall (RF) irrigation conditions. Abbreviations: TPC, Total Phenolic Content; GAE, Gallic Acid Equivalents; TE, Trolox Equivalents, H, Hydrolysate; H-D, Hydrolysate Digested. Different lowercase letters indicate significant differences in the mean values (one-way ANOVA, Duncan’s test, *p* ≤ 0.05) between samples.

The impact of simulated gastrointestinal digestion on the antioxidant properties of a hemp seed–derived ingredient from the Henola cultivar was evaluated (Figure 6). Antioxidant parameters were analysed before and after in vitro digestion through five complementary assays: total phenolic content, oxygen radical absorbance capacity, ABTS^●+^ radical scavenging activity, DPPH^●^ radical scavenging activity, and ferric reducing antioxidant power. Pre-digestion (light bars) and post-digestion (dark bars) values were compared, and statistical significance was assessed using one-way ANOVA followed by Duncan’s test (*p* ≤ 0.05).

Across all assays, a consistent and statistically significant decrease in antioxidant values was observed following digestion, indicating that gastrointestinal conditions substantially reduce the measurable antioxidant capacity and phenolic content of the hemp seed sample. TPC and DPPH^●^ were the parameters that exhibited the most pronounced reductions, suggesting that a substantial fraction of phenolic compounds and radical-scavenging capacity becomes less bioaccessible after digestion. ORAC, ABTS^●+^, and FRAP values also decreased but retained residual activity, indicating that some antioxidants persist after the digestion process and remain potentially bioaccessible.

These findings align with published research on hemp seed bioactives and nutraceutical potential. For example, metabolomic profiling study of seven hemp seed varieties identified over 1000 metabolites—including 201 flavonoids and 149 phenolic acids—and demonstrated that these compounds, together with free fatty acids and terpenes, strongly correlate with antioxidant activity [45]. The persistence of residual antioxidant capacity post-digestion in the present study suggests that these metabolite classes may represent the bioaccessible fraction.

Furthermore, investigations into hemp seed-derived nutraceutical ingredients have emphasized the challenges of extraction, stability, and bioaccessibility. For instance, research on cannabidiol (CBD) nanoemulsions formulated with hemp seed oil demonstrated improved bioaccessibility of CBD compared to bulk oil, underscoring the importance of formulation in enhancing compound availability [54]. Such strategies could help mitigate the digestion-induced reduction in antioxidant activity observed here.

Additionally, comparisons between whole and dehulled hemp seeds have shown that whole seeds generally possess higher phenolic content and antioxidant activity, likely due to the presence of phenolics in the hull [55]. This indicates that seed matrix and processing—such as dehulling or solvent extraction—can influence antioxidant yield and, consequently, the fraction that survives digestion and remains bioaccessible.

Taken together, these results indicated that although hemp seeds are a rich source of phenolic and antioxidant compounds, gastrointestinal digestion significantly reduced the fraction that remained extractable and active. For nutraceutical applications, this underscores the need to develop formulations and extraction processes that preserve or release bioactive compounds in forms that remain stable and bioaccessible post-digestion. Approaches such as encapsulation, nanoemulsion systems, or the use of more resilient seed derivatives may improve post-digestive bioavailability [56,57]. Overall, while hemp seed represents a promising source of functional bioactives, optimizing bioaccessibility after digestion is essential for achieving nutraceutical efficacy.

### 3.7. In Vitro Digestion of Nutraceutical Gummy Candy

**Figure 7:**
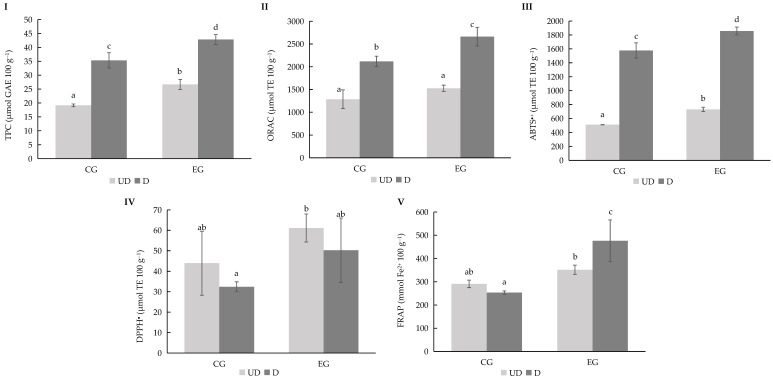
TPC (**I**), ORAC (**II**), ABTS^●+^ (**III**), DPPH^●^ (**IV**), and FRAP (**V**) values for *Cannabis sativa* L. Henola variety cultivated under rainfall (RF) irrigation conditions. Abbreviations: TPC, Total Phenolic Content; GAE, Gallic Acid Equivalents; TE, Trolox Equivalents; CG, Control Gummy; EG, Enriched Gummy; UD, Undigested; D, Digested. Different lowercase letters indicate significant differences in the mean values (one-way ANOVA, Duncan’s test, *p* ≤ 0.05) between samples.

The gummies incorporating the hydrolysed ingredient were designed to evaluate the feasibility of a incorporating bioactive ingredients into a nutraceutical product. The comparison with gummy control formulations showed that the inclusion of hydrolysate did not significantly change (*p* ≤ 0.05) the macronutrient balance of the confection, with water, sugar, pectin, and lemon juice remaining the primary constituents (Table 1). The hydrolysate represented only a small percentage of the total formulation without impact from a nutritional point of view. However, the chemical characterization of gummy candy confirmed the presence of phenolic compounds and antioxidant activity in the hydrolysate-enriched samples. Compared to the control, fortified gummy exhibited higher total phenolic content and significantly improved antioxidant properties, particularly in assays sensitive to radical scavenging and reducing power. It was interesting that these differences were maintained after the confectionery process, suggesting that the bioactive compounds from hemp hydrolysate were stable under the thermal and physical conditions involved in gummy preparation. This highlights the stability of hemp-derived antioxidants during food processing (Figure 7).

Similar stability of phenolics during processing has been reported for fortified confections made with grape seed and green tea extracts, where functional compounds were retained after gelatin-based formulation [58]. Likewise, fruit-based gummies enriched with plant polyphenols showed sustained antioxidant performance, supporting their use as delivery matrices for bioactive ingredients [59]. The observed stability suggests that hemp hydrolysate can be successfully embedded in a consumer-friendly format without compromising efficacy, reinforcing the role of gummy candies as suitable carriers of functional ingredients for nutraceutical applications.

These results agree with previous findings on polyphenol-rich foods, where digestion often reduces total phenolics due to breakdown, transformation, or interaction with digestive components [60]. However, the fact that enriched gummy candies maintained significantly higher post-digestion activity reflects the robustness of hemp-derived bioactives. Comparable studies with berry-enriched gummies demonstrated that although phenolic content decreased after digestion, fortified products still provided meaningful antioxidant activity [61]. This indicates that despite partial losses during digestion, hemp hydrolysate can enhance the functional performance of nutraceutical gummy candy, supporting its bioactive potential in vivo.

### 3.8. ACE Inhibitory Activity of Nutraceutical Gummy Candy

The study of angiotensin-converting enzyme (ACE) inhibition showed that hemp hydrolysate had a strong antihypertensive potential, with activity exceeding that of both the control and hydrolysate-enriched gummy candy. The dose–response curves (Figure S1) showed that inhibition increased progressively with concentration, and hydrolysate alone achieved the highest inhibition rates. Incorporation of the hydrolysate into the gummy did not significantly improve ACE inhibitory activity. On the other hand, digestion improved both sample (CG and EG) IC_50_ results, with slightly higher, although not significantly (*p* ≤ 0.05), ACE inhibitory capacity, in the case of hydrolysate-enriched gummy candy.

These observations were consistent with previous reports that hemp protein hydrolysates generate peptides with potent ACE inhibitory activity [62,63]. Similar findings have been reported in other oilseed proteins, such as soy and rapeseed, where hydrolysis released antihypertensive peptides that retained activity under simulated gastrointestinal conditions [64]. The partial attenuation of activity in gummy candies parallels prior evidence that the confectionery matrix can limit peptide release and bioavailability [65]. Nonetheless, the presence of sustained inhibition highlights hemp hydrolysate as a promising nutraceutical ingredient for blood pressure management.

The regression analysis of ACE inhibition curves (Figure S1, Table S1) yielded high coefficients of determination, indicating consistent and reliable concentration–response relationships. IC_50_ values clearly distinguished the hydrolysate as the most effective treatment, with much lower values compared to gummy candy formulations and controls. The digested hydrolysate maintained the strongest inhibitory capacity, while digested gummy candies showed intermediate activity.

**Figure 8:**
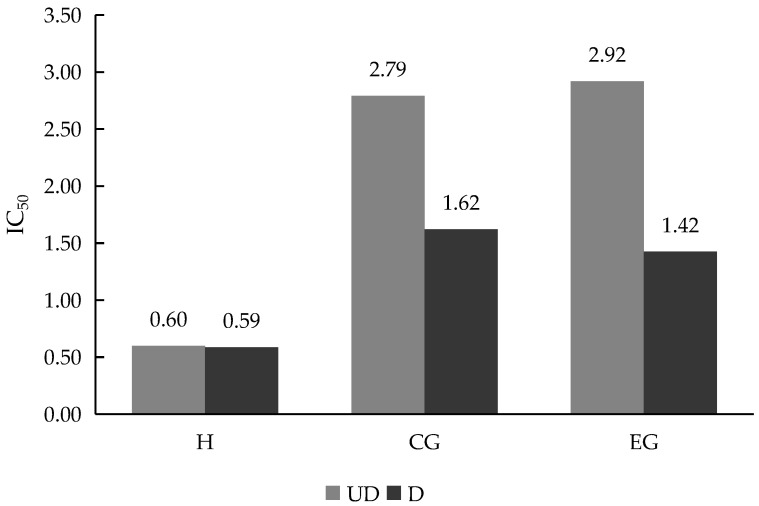
IC50 values of hydrolysate and gummy samples. Abbreviations: H, hemp hydrolysate; CG, control gummy; EG, enriched gummy; UD, undigested; D, digested.

These results reinforce earlier studies in which hemp protein hydrolysates demonstrated IC_50_ values comparable to those of peptides from soy and caseins [66]. The finding that digestion preserved or even enhanced inhibitory potential is in line with the work by Udenigwe & Aluko [67], who reported that gastrointestinal enzymes often generate smaller peptides with stronger ACE inhibitory activity than the original hydrolysates. In the current study, the significant (*p* ≤ 0.05) differences between treatments emphasize the importance of delivery format and digestion in shaping antihypertensive bioactivity (Figure 8).

The final comparison of IC_50_ values across all treatments confirmed the superior performance of hemp hydrolysate, both undigested and digested, in inhibiting ACE. Gummy candy enriched with hydrolysate showed IC_50_ values similar to those of control formulations. Digestion did not change hydrolysate ACE inhibitory activity, while gummy candy was positively affected, showing improved performance relative to undigested samples. Although the differences in digested gummies (EG and EG) were not significant, the change in trend as compared with undigested samples may be due to an effect of hydrolysate in the formula (tested concentration was relatively low, at 0.66 g of ingredient in 100 g of gummy). For this reason, increased extract concentrations could enhance this activity.

Comparable outcomes have been reported in studies where plant protein hydrolysates were incorporated into functional foods; although the matrix moderated peptide release, measurable ACE inhibitory activity remained [68]. The persistence of antihypertensive potential after digestion highlights hemp hydrolysate as a bioactive ingredient of clinical interest, aligning with the growing literature on dietary peptides as natural alternatives or complements [69]. These findings underline both the opportunities and challenges of translating bioactive extracts into consumer products without compromising efficacy.

### 3.9. Textural Properties of Gummy Candy

**Figure 9:**
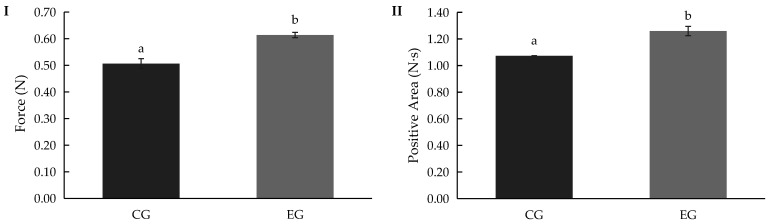
Maximum force (**I**) and positive area (**II**) values of gummy samples. Abbreviations: CG, control gummy; EG, enriched gummy. Different lowercase letters indicate significant differences in the mean values (one-way ANOVA, Duncan’s test, *p* ≤ 0.05) between samples.

Figure 9 shows the texture parameters obtained from the compression test of the gummy formulations. The maximum force (I) represents the resistance to deformation, while the positive area (II) reflects the energy required to compress the gummy. The enriched gummy (EG) showed values that were comparable to or slightly higher than those of the control gummy (CG), suggesting that the incorporation of the bioactive hydrolysate did not compromise the mechanical integrity of the product. These findings confirmed that the nutraceutical ingredient can be successfully incorporated into gummy formulations without altering their desirable texture characteristics.

Figure S1 presents the profile compression curves obtained for the control and enriched gummies. The similar curve behaviour between CG and EG supports the conclusion that the addition of the hydrolysate did not induce structural heterogeneity or excessive stiffness in the gummy matrix. Each profile corresponds to one of the three independent replicates. The overlapping patterns and consistent peak profiles indicate a high reproducibility of the texture measurements.

### 3.10. Sensory Properties of Gummy Candy

**Figure 10:**
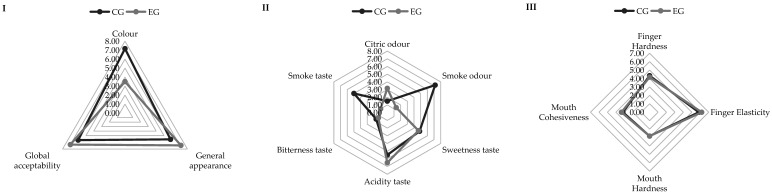
Appearance and global acceptability (**I**), olfactory-gustatory (**II**) and texture sensory analysis (**III**) values of gummy samples. Abbreviations: CG, control gummy; EG, enriched gummy.

The sensory comparison between control (CG) and enriched gummies (EG) showed that visual attributes were largely maintained following enrichment. As shown in Figure 10(I), both formulations achieved comparable scores for global acceptability and general appearance, indicating that the incorporation of bioactive or functional ingredients did not negatively influence consumer perception. The enriched formulation, however, presented slightly higher colour intensity values, which may be associated with the presence of natural pigments or extracts used in the enrichment process. Similar findings have been reported for fortified confectionery systems, where the addition of fruit- or plant-derived compounds enhances visual appeal without compromising overall acceptance [70].

Differences were more evident in the odour and taste attributes (Figure 10(II)). The enriched gummies exhibited higher citric odour and acidity scores, together with reduced sweetness and bitterness perception compared to the control. These sensory shifts may be linked to the chemical composition of the added ingredients, which can introduce acidic or aromatic compounds and partially mask sweetness. The predominance of acidic and fruity notes suggests a more complex flavour profile typical of formulations containing polyphenol- or vitamin-rich additives [71]. Such modifications are consistent with reports showing that enrichment with functional ingredients can alter flavour balance through interactions with sugars, acids, and flavouring agents [72].

Textural analysis (Figure 10(III)) indicated that enrichment slightly increased hardness and elasticity, both in finger and mouth evaluations. These findings agreed with the results obtained using instrument analysis (Figure 9 and Figure S1). This behaviour may result from the reinforcement of the gel network structure, likely due to interactions between gelling agents (such as gelatin or pectin) and the bioactive compounds introduced during enrichment. Improved cohesiveness and elasticity have been similarly observed in fibre- or protein-fortified gummies, reflecting enhanced water-binding and crosslinking within the matrix [73]. Overall, the results demonstrate that enrichment modified the sensory profile—particularly taste and texture—without compromising the general acceptability of the gummies.

## 4. Conclusions

In conclusion, this study demonstrates the significant influence of genetic and agronomic practise and bioprocessing on the nutritional, phytochemical, and functional properties of *Cannabis sativa* L. seeds and their derived nutraceutical products. Genotype and water availability were major determinants of macronutrient composition in the three hemp cultivars (Futura 75, Henola, and KC Zuzana), with irrigation enhancing lipid and protein content in KC Zuzana and Henola, while Futura 75 exhibited higher carbohydrate and fibre levels under rainfall conditions. These findings highlight the importance of selecting cultivars not only for nutritional traits but also based on phytochemical profiles, such as CBD:THC ratios, to ensure consistent bioactive content.

Phenolic content and antioxidant activity were also affected by genotype and water management, with rainfall increasing total phenolics and antioxidant capacity, particularly in Henola, consistent with stress-induced phenolic biosynthesis. Enzymatic hydrolysis of Henola seeds improved bioaccessibility by breaking down cell wall–phenolic complexes, enhancing antioxidant activity, and providing insight into potential antihypertensive mechanisms, including ACE inhibition probably associated with the increase in bioaccessible phenolic and eptides released during the process.

Simulated gastrointestinal digestion reduced phenolic content and antioxidant activity, although residual bioactivity persisted across ABTS^●+^, FRAP, ORAC, and DPPH^●^ assays, suggesting that hemp seed phenolics and peptides remain bioaccessible in vivo. These observations underscore the need for pharmacokinetic studies to evaluate absorption, distribution, metabolism, and excretion (ADME) of hemp-derived gummies. The hydrolysate exhibited strong ACE inhibitory activity, which was retained post-digestion, supporting its potential blood pressure-modulating effects.

Incorporation of the hydrolysate into a gummy matrix preserved its antioxidant properties and phenolic content, while maintaining structural integrity and acceptable sensory characteristics. The enriched gummies demonstrated enhanced antioxidant capacity without negatively affecting texture or flavour, likely due to stabilizing interactions between pectin and bioactives.

Overall, this study confirms that hemp hydrolysates can be successfully formulated into nutraceutical gummies while maintaining functional and sensory quality. Future research should focus on establishing safe and effective dosage levels through controlled studies, monitoring long-term intake and potential side effects, to optimize the antihypertensive benefits of hemp-enriched gummies.

## Figures and Tables

**Table 1:** Gummy candy formulation expressed in grams per 100 g and per gummy candy.

	Control Gummy		Hydrolysed Gummy	
	Grams per 100 g	Grams per Gummy	Grams per 100 g	Grams per Gummy
**Water**	45.956	0.919	45.653	0.913
**Agave syrope**	22.978	0.46	22.826	0.457
**Pectin**	1.608	0.032	1.598	0.032
**Sacarose**	11.029	0.221	10.957	0.219
**Lemon juice**	18.382	0.368	18.261	0.365
**Ginger**	0.046	0.001	0.046	0.001
**Hemp hydrolysed ingredient**	−	−	0.66	0.013

**Table 2:** Proximal composition (grams per 100 g^−1^) for hemp seeds.

	Futura 75		Henola		KC Zuzana	
	RF	RFCI	RF	RFCI	RF	RFCI
**Ash**	5.05 ± 0.29 ^ab^	6.23 ± 0.35 ^c^	4.39 ± 0.25 ^a^	5.28 ± 0.30 ^b^	4.43 ± 0.25 ^a^	7.57 ± 0.43 ^d^
**Fat**	19.50 ± 1.10 ^a^	34.70 ± 1.96 ^d^	23.87 ± 1.35 ^b^	28.86 ± 1.63 ^c^	24.12 ± 1.36 ^b^	36.29 ± 2.05 ^d^
**TDF**	53.49 ± 6.81 ^b^	28.23 ± 3.59 ^a^	48.91 ± 6.22 ^b^	35.03 ± 4.46 ^a^	50.26 ± 6.40 ^b^	30.33 ± 3.86 ^a^
**Moisture**	6.15 ± 0.35 ^a^	5.77 ± 0.33 ^a^	6.15 ± 0.35 ^a^	6.09 ± 0.34 ^a^	5.89 ± 0.33 ^a^	6.03 ± 0.34 ^a^
**Protein**	19.71 ± 1.12 ^a^	29.18 ± 1.65 ^c^	19.65 ± 1.11 ^a^	29.42 ± 1.66 ^c^	20.86 ± 1.18 ^ab^	23.61 ± 1.34 ^b^
**Carbohydrates**	55.74 ± 3.94 ^b^	29.88 ± 2.11 ^a^	52.09 ± 3.68 ^b^	36.44 ± 2.58 ^a^	50.59 ± 3.58 ^b^	32.53 ± 2.30 ^a^

**Abbreviations:** Rainfall (RF) and rainfall and conventional irrigation conditions (RFCI), total dietary fibre (TDF). Different lowercase letters in the same row indicate significant differences in the mean values (one-way ANOVA, Duncan’s test, *p* ≤ 0.05) between samples.

## Data Availability

The datasets generated and/or analysed during the current study are not publicly available because the institute did not have an open repository, but are available from the corresponding author on reasonable request.

## Supplementary Materials

The following supporting information can be downloaded at: https://www.mdpi.com/article/10.3390/bioengineering12121298/s1, Table S1: Linear regression equation and R^2^ values of control gummy (**I**), enriched gummy (**II**), hemp hydrolysate (**III**), control gummy digested (**IV**), enriched gummy digested (**V**), hemp hydrolysate digested (**VI**); Figure S1: ACE % inhibition graphs and IC_50_ values of control gummy (**I**), enriched gummy (**II**), hemp hydrolysate (**III**), control gummy digested (**IV**), enriched gummy digested (**V**), hemp hydrolysate digested (**VI**); Figure S2: Force versus time graph of gummy samples.
