# Supplementary material for: Design of a Nutraceutical Gummy Candy Incorporating Hydrolysed Hemp (Cannabis sativa L.) as an Antioxidant and Antihypertensive Ingredient
Source: Bioengineering (Basel). 2025 Nov 25;12(12):1298. doi: 10.3390/bioengineering12121298 (PMC12729579; doi:10.3390/bioengineering12121298)
Supplement: Supplementary file 1 [file bioengineering-12-01298-s001.zip › bioengineering-3997606-supplementary.pdf]

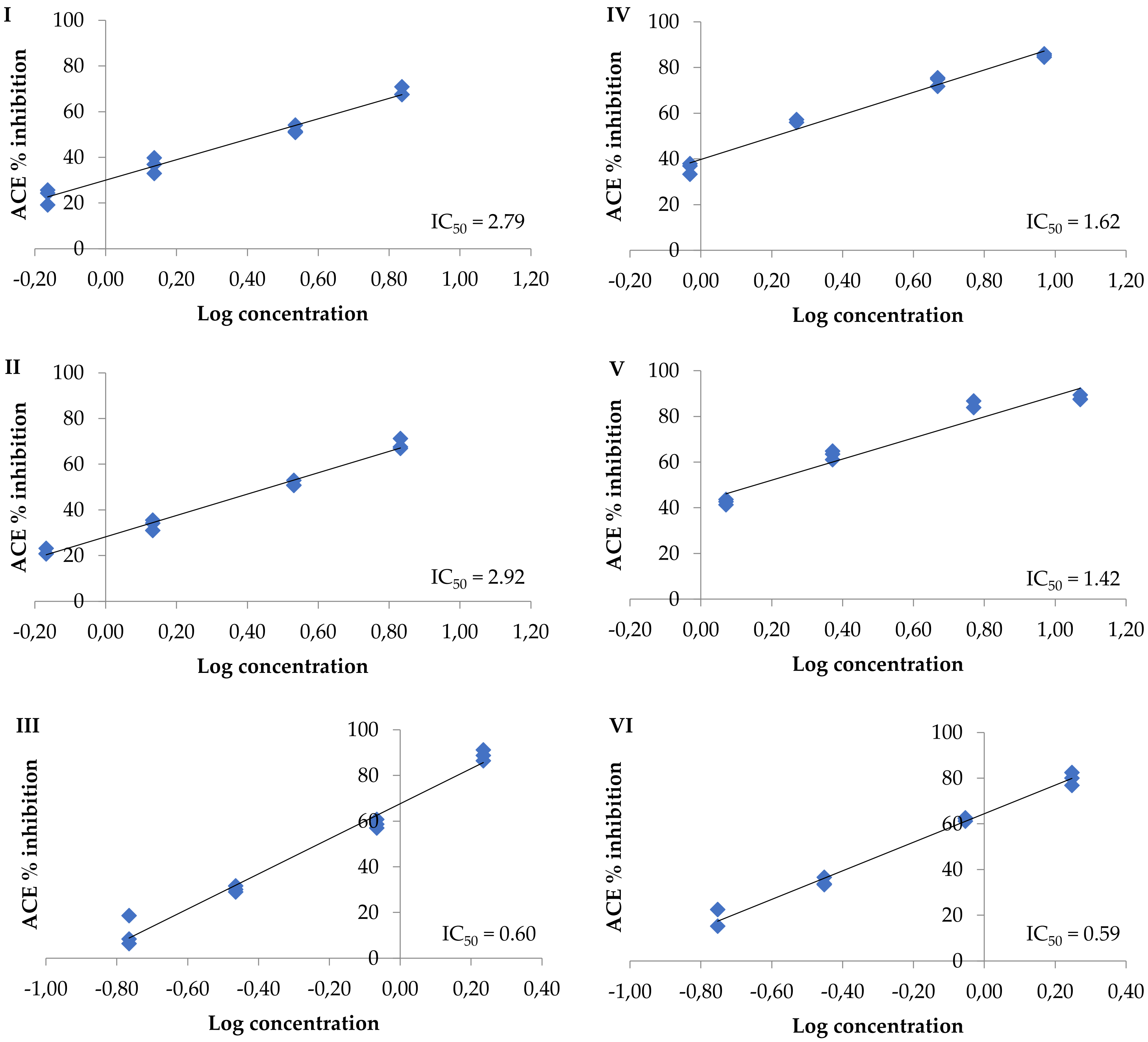

Figure S1. ACE % inhibition graphs and IC_{50} values of control gummy (**I**), enriched gummy (**II**), hemp hydrolysate (**III**), control gummy digested (**IV**), enriched gummy digested (**V**), hemp hydrolysate digested (**VI**)

Table S1. Linear regression equation and R^2 values of control gummy (**I**), enriched gummy (**II**), hemp hydrolysate (**III**), control gummy digested (**IV**), enriched gummy digested (**V**), hemp hydrolysate digested (**VI**)

	**Linear regression equation**	**R^2**
I	y = 44.718x + 30.059	0.9787
II	y = 46.78x + 28.231	0.9873
III	y = 76.827x + 67.602	0.9804
IV	y = 48.978x + 39.73	0.9802
V	y = 46.195x + 42.897	0.9395
VI	y = 62.453x + 64.449	0.9889

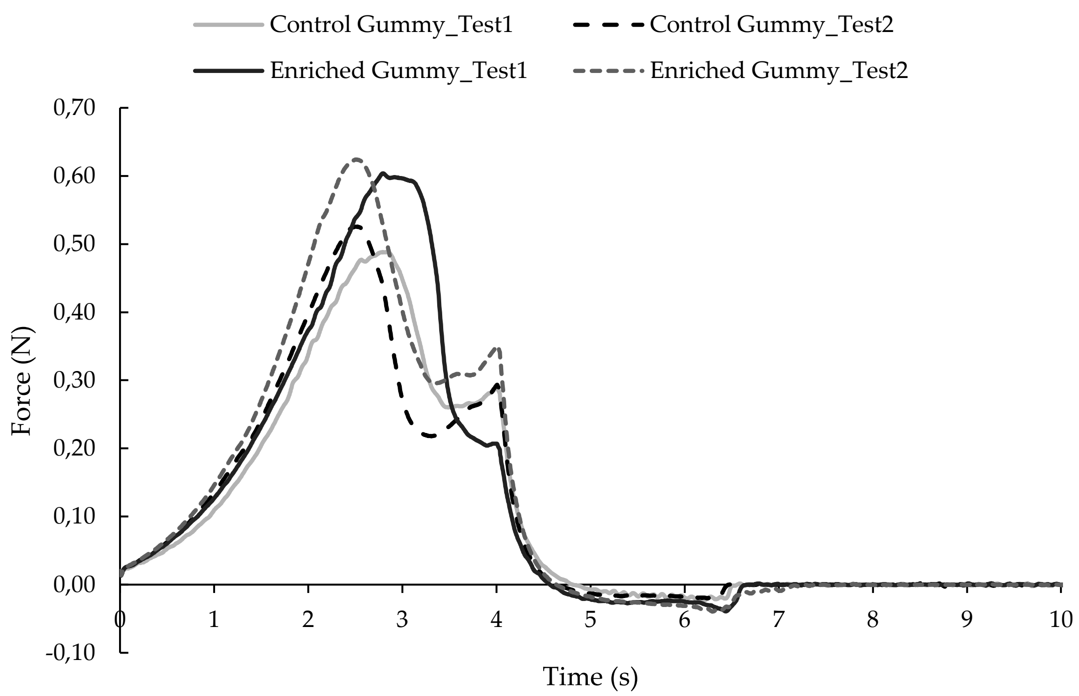

Figure S2. Force versus time graph of gummy samples
